# Accident Vulnerability and Vision for Action: A Pilot Investigation

**DOI:** 10.3390/vision4020026

**Published:** 2020-05-13

**Authors:** Anthony J. Lambert, Tanvi Sharma, Nathan Ryckman

**Affiliations:** School of Psychology and Centre for Brain Research, University of Auckland, Auckland 1010, New Zealand; tanvi.sharma@auckland.ac.nz (T.S.); n.ryckman@auckland.ac.nz (N.R.)

**Keywords:** vision for action, accidents

## Abstract

Many accidents, such as those involving collisions or trips, appear to involve failures of vision, but the association between accident risk and vision as conventionally assessed is weak or absent. We addressed this conundrum by embracing the distinction inspired by neuroscientific research, between vision for perception and vision for action. A dual-process perspective predicts that accident vulnerability will be associated more strongly with vision for action than vision for perception. In this preliminary investigation, older and younger adults, with relatively high and relatively low self-reported accident vulnerability (Accident Proneness Questionnaire), completed three behavioural assessments targeting vision for perception (Freiburg Visual Acuity Test); vision for action (Vision for Action Test—VAT); and the ability to perform physical actions involving balance, walking and standing (Short Physical Performance Battery). Accident vulnerability was not associated with visual acuity or with performance of physical actions but was associated with VAT performance. VAT assesses the ability to link visual input with a specific action—launching a saccadic eye movement as rapidly as possible, in response to shapes presented in peripheral vision. The predictive relationship between VAT performance and accident vulnerability was independent of age, visual acuity and physical performance scores. Applied implications of these findings are considered.

## 1. Introduction

Although vision-for-action is a well-established concept in basic visual neuroscience [1,2], applied implications of this aspect of visual functioning have yet to be explored in depth. Here, we report a preliminary investigation using a novel technique for assessing individual differences in vision for action, and show that scores on our ‘Vision for Action Test (VAT)’ are related to a self-report measure of accident vulnerability, independently of visual acuity, age, or the ability to carry out physical actions that involve balance, standing and walking.

Failure to link vision with action is a key causal factor in many personal accidents. For example, failure to link visual input with motor systems is likely to contribute to accidents that involve tripping, falling or colliding with objects [3]. Moreover, failure to respond appropriately to visual information by shifting attention, either overtly by moving the eyes, head or body, or covertly [4], contributes to a large proportion of motor vehicle accidents involving inattention [5,6]. These considerations might lead one to expect a clear association between visual functioning and accident vulnerability. Disappointingly, the relationship between conventional tests of vision and the risk of being involved in a motor vehicle accident is weak or non-existent [7,8]. Although the absence of a clear relationship between conventional tests of vision and accident vulnerability seems surprising at first blush, it is less so when viewed in the context of dual-stream models of vision inspired by neuroscientific research. According to the influential model proposed by Milner and Goodale [2] vision arises from processing operations in two distinct, but interacting cortical pathways, together with subcortical contributions. The ventral stream is a network of visually responsive areas linking primary visual cortex (V1) with inferotemporal cortex, while the dorsal stream is a network of interconnected visual areas linking V1 with posterior parietal cortex. According to Milner and Goodale [2] the ventral stream delivers conscious experience of the visual world—vision for perception—while the dorsal stream carries out the rapid on-line computations necessary for performing visually guided actions, such as locomotion, avoiding obstacles, reaching and grasping. That is, the dorsal stream delivers vision-for-action. Although not without critics [9], dual-stream characterisations of visual functioning have been supported by an impressive array of findings from research with animal [10] as well as human [2,11] participants, including neuropsychological, behavioural and neuroimaging [12,13] studies. In extending this framework, Lambert et al. [14] noted that shifts of attention, both overt and covert, can be considered visually guided actions, and presented evidence in support of the proposal that rapid shifts of attention are associated with dorsal stream encoding of visual input (see also [15,16]). Viewed from this dual-stream perspective, it seems likely that vision-for-action (including the ability to shift attention rapidly in response to new visual information), rather than vision-for-perception, is likely to be the aspect of visual functioning most closely associated with accident vulnerability. This proposal does not, of course, exclude the possibility that some accidents are linked with ventral stream functioning. Moreover, under normal circumstances, the dorsal and ventral stream interact closely with each other, and operate as a well-integrated whole. A failure of dorsal-stream-mediated visual orienting, such as failing to orient attention towards a hazardous object, will have a down-stream consequence for ventral stream processing—the hazardous object will fail to access vision-for-perception, and fail to achieve conscious representation. Hazards may also fail to be perceived due to peripheral sensory failures, caused by conditions such as macular degeneration or uncorrected myopia. Having acknowledged these caveats, our central hypothesis is a direct corollary of the proposal of Milner and Goodale [2] that vision-for-action enables rapid and accurate performance of visually guided actions: Accordingly, sub-optimal vision for action should be associated with impaired performance of visually guided actions, and increased susceptibility to accidents caused by failures to link vision with action. Accordingly, our hypothesis predicts that self-reports of accident vulnerability will be associated with individual differences in vision for action. Below we introduce a new self-report measure of accident vulnerability—the Accident Proneness Questionnaire (APQ)—and explain a novel technique for assessing individual differences in vision for action—the Vision for Action Test (VAT).

A key feature of the Milner and Goodale [2] framework is that, while the processing operations of the ventral visual stream are associated with conscious perceptual experience, processing within the dorsal stream is thought to be non-conscious, and indeed inaccessible to consciousness [13]. Classical psychophysics and techniques for assessing vision in optometric settings rely almost exclusively on conscious decisions and reports about visual stimuli. Moreover, because ‘vision’ tends to be identified with ‘conscious seeing’, the assumption that asking an individual what they can see is the only viable approach for measuring visual functioning is widespread. However, if one accepts the dual-stream framework just described, it is clear that comprehensive assessment of visual functioning requires the development of alternative approaches, which assess the efficiency of non-conscious visual processing by the dorsal stream [17]. In the current study, we met this challenge by embracing Milner and Goodale’s [1,2] description of dorsal stream function in terms of vision-for-action, and assessed the efficiency with which participants could perform a specific kind of visually guided action—moving the eyes in response to a peripherally presented landmark stimulus (see [14]). We predicted that individual differences in vision for action would be related to accident vulnerability, independently of a conventional measure of vision for perception—the Freiburg Vision Test [18], which assesses visual acuity.

Hence, the ability to direct an eye movement appropriately in response to peripheral visual information was the key behaviour assessed in our Vision for Action Test (VAT). This novel test incorporates design features that target dorsal visual stream processing. Its structure is based on the landmark cueing procedure [14,15,19,20] which combines elements of the attentional cueing techniques devised by Michael Posner and colleagues [4,21] and the landmark learning procedure described by Ungerleider and Mishkin [10]. In landmark cueing, participants shift attention towards a peripheral target object, which is preceded by bilateral cue stimuli. The target object is likely to appear at the same location as one of the cues, termed the landmark stimulus. Evidence from a number of studies indicates that the dorsal visual stream plays a key role in the featural encoding that drives rapid attention-shifting in response to landmark cues. These include: (1) studies showing that behavioural characteristics of orienting in response to landmark cues correspond with known physiological characteristics of the dorsal visual stream, including sensitivity to low contrast edges, representation of the visual periphery and insensitivity to isoluminant stimuli [14,20,22]; (2) high-density EEG studies, in which source localization has indicated early activation of dorsal stream structures following presentation of landmark cues [15,16,19]; and (3) neuropsychological studies showing that a patient (DF), with bilateral damage to the ventral stream and dense visual-form agnosia was able to shift attention in response to landmark cues, despite being unable to discriminate consciously between alternative cue stimuli [16]. In contrast, a patient with a dorsal stream (parietal–occipital) lesion and optic ataxia was unable to shift attention in response to landmark cues but retained the ability to shift attention in response to symbolic cues [23]. Therefore, our proposal that the dorsal visual stream carries out visual-spatial encoding that supports orienting in response to landmark cue features is buttressed by substantial converging evidence from a number of sources.

In the Vision for Action Test described in detail below, participants were presented with peripheral shapes, comprising a circle and a triangle, and were asked to move their eyes to the location of the landmark shape (circle or triangle, counter-balanced between participants), in order to discriminate a target object (a digit) presented there. Participants were required to decide whether the digit was 2 or 7. The stimulus onset asynchrony (SOA) between onset of the peripheral shape cues, and onset of the target object was gradually reduced, via a staircase procedure [24], until the accuracy of eye movement and target discrimination decisions reached a stable threshold value of 75% correct. A participants’ VAT threshold score was the average SOA value, across two repetitions of this procedure. Hence, lower VAT scores represent better performance, with respect to launching an eye movement in response to information provided by the shape cues.

It is worth noting that, in VAT, the feature chosen for staircase manipulation to establish a threshold is temporal. The rationale for this was based on the pre-eminence of the temporal dimension in theoretical descriptions of dorsal stream encoding. That is, according to the framework proposed by Milner and Goodale [1,2], the dorsal stream is specialised for the rapid encoding necessary for moment-to-moment, on-line control of visually guided actions. The rapidity of dorsal stream visual encoding was also emphasised by Bullier [25], who describes the parietal targets of dorsal stream encoding as “the fast brain”.

Clear associations have been noted between age and dorsal stream functioning [26], and also between age and accident vulnerability [27,28,29,30]. Accordingly, it was important to establish that any association between accident vulnerability and our measure of vision for action cannot simply be attributed to correlations of both these factors with a further variable—age. Moreover, a longer-term goal of our work is to investigate whether vulnerability to accidents among older adults can be mitigated through interventions targeting vision for action. However, establishing whether there is indeed an association between vision for action and accidents is a necessary prerequisite for this avenue of research and application. In light of these considerations, the current study was carried in two successive stages. During Stage One, samples of older and younger participants completed a self-report measure of accident vulnerability—the Accident Proneness Questionnaire (APQ). Stage One participants also completed questionnaires assessing self-reported cognitive failures (Cognitive Failures Questionnaire: [31]), and concerns about falling while carrying out everyday activities, such as taking a bath or shower (Falls Efficacy Scale-international (FES-I); [32,33]). In Stage Two, smaller samples of participants with relatively high and relatively low APQ scores, in both age groups, were invited to return to the laboratory, in order to complete the Vision for Action Test. During this stage, participants also performed a test of visual acuity (Freiburg Vision Test; [18]), and a measure of physical performance and gait (Short Physical Performance Battery; [34]. Lauretani et al. [35] found that SPPB scores predicted the likelihood of experiencing a particular kind of accident—falling. This two-stage strategy ensured that our final sample included younger and older participants, and individuals with low and high self-reported accident vulnerability within each age group, enabling links between vision for action and accident vulnerability to be disentangled from effects of age on both these constructs. Moreover, our design also enabled links between APQ and Short Physical Performance Battery scores to be evaluated.

## 2. Method (Stage One)

**Participants.** Twenty-eight healthy older (aged 65 and over) and 14 younger volunteers aged 18–30 took part. All participants provided informed consent; the procedures were approved by the University of Auckland Human Participants Ethics Committee (Approval number: 021042).

**Design and procedure.** Each participant completed three self-report questionnaires: the Accident Proneness Questionnaire (APQ—see Appendix A), Cognitive Failures Questionnaire (CFQ; [31]) and Falls Efficacy Scale-international (FES-I; [32,33]).

The APQ is a 10-item scale which assesses the self-reported frequency of accidents, including falls, bumping into obstacles, dropping things, road accidents and road accident near-misses (see Appendix A for full details, and scoring rubric). The CFQ [31] is a 25-item questionnaire which assesses the self-reported frequency of everyday cognitive errors, such as forgetting where you put something, or failing to notice signposts on the road. The FES-I is a 16-item scale which assesses the extent to which an individual is concerned about falling while carrying out everyday activities, such as walking around the neighbourhood, taking a bath or shower, or walking on a slippery surface (e.g., wet or icy).

Participants responded to APQ items using a Likert scale with values ranging from 1 to 5 (see Appendix A). The APQ included items such as “Do you hurt yourself as a result of bumping into objects such as furniture, doors or lamp posts?”, and “When crossing the road, do you sometimes fail to notice an approaching vehicle and begin to cross, when you should have waited?” Higher APQ scores reflect greater accident proneness. Participants responded to FES-I using a four-point Likert scale (from 1 = “not at all concerned” to 4 = “very concerned”). FES-I includes items such as “How concerned are you about falling when getting in or out of a chair” (see [32]). Participants responded to CFQ using a five-point Likert scale (from 0 = “never” to 4 = “very often”) and included questions such as “Do you forget why you went from one part of the house to the other” (see [31]).

## 3. Results (Stage One)

One participant failed to complete the FES-I inventory. Mean APQ, CFQ and FES-I scores for older and younger participants are shown in Table 1, and bivariate correlations between APQ, CFQ and FES-I scores are shown in Table 2.

In addition, the internal consistency of the 10 items of the APQ was evaluated. Since none of the forty-two participants had experienced an accident as a pedestrian in the past five years, the variance of Item 7 was zero, and therefore this item was excluded. Cronbach’s alpha for the remaining nine items was 0.69. Values for Cronbach’s alpha are known to be influenced positively by the number of items in a scale [36,37]. Accordingly, in light of the small number of items examined (*N* = 9), this value was considered acceptable. Moreover, some authors [38] consider the mean inter-item correlation to be a better reflection of consistency than Cronbach’s alpha. Mean inter-item correlation for the APQ (excluding Item 7) was *r* = 0.17, a value within the range (0.15–0.50) recommended by Clark and Watson [38]. (In a follow-up study [39], the internal consistency of a revised 9-item version of the APQ was studied in a larger sample (*N* = 411). Internal consistency of the revised APQ was acceptable (Cronbach’s alpha = 0.70; mean inter-item correlation was *r* = 0.25).

## 4. Discussion (Stage One)

As Table 1 shows, the samples of older and younger individuals who volunteered to participate in Stage One did not differ reliably with respect to accident vulnerability, cognitive failures, or self-reported concerns about falling. As noted in our introduction, one might have expected the older group to report greater accident vulnerability, more concern about falling, and possibly more frequent cognitive failures (but see [40]). However, as Table 1 shows, this was not the case. Hence, the older adults who volunteered to participate in this study appeared to be functioning very well with respect to these constructs, and at a level comparable to the younger participants.

Table 2 shows, perhaps unsurprisingly, that self-reports of accidents were correlated reliably with concerns about a specific kind of accident—falling, reflected in FES-I scores. APQ scores were also correlated with everyday cognitive failures. This relationship might also be expected, because, like the APQ, the CFQ inventory contains items reflecting failures of visually guided action (“Do you bump into people?”), as well as items reflecting attentional failure (“Do you fail to notice signposts on the road?”). Prior to undertaking this study, a self-report scale comprising items targeted specifically at the vision for action functions associated with the dorsal visual stream was not available. Therefore, the items of the APQ were developed specifically to fulfil this purpose. While further investigation of the psychometric properties of the APQ would clearly be valuable, the primary purpose of the initial stage of this investigation was to identify older and younger participants with relatively high and relatively low accident vulnerability. This strategy ensured that testing for the predicted association between APQ scores and vision for action would not be compromised by an adventitiously narrow range of APQ scores in our sample. Moreover, as noted earlier, we aimed to disentangle influences of age on both vision for action and accident vulnerability, by including participants with relatively high and relatively low APQ scores within each age group.

## 5. Method (Stage 2)

**Participants.** Nine younger participants and eight older participants scoring high and low on APQ volunteered to participate in Stage 2. All participants provided informed consent, and the procedures were approved by the University of Auckland Human Participants Ethics Committee. (Approval number: 021042). Data from three younger participants was unusable, due to difficulty in calibrating the eye tracker during VAT administration. Therefore the final sample comprised six younger participants (three scoring high (mean = 22.9) and three scoring low (mean = 16.0) in APQ) and eight older participants (four scoring high (mean = 23.8) and four scoring low (mean = 16.5) in APQ). Mean age of the younger group was 22.5 years (SD = 1.4); mean age of the older group was 76.4 (SD = 2.5).

**Apparatus.** The Short Physical Performance Battery (SPPB; [34]) required a scoring sheet and a stopwatch. The Freiburg Vision Test used a Dell Optiplex PC with 23” LED monitor. Viewing distance from the screen was 4 m and participants responded to Landolt C optotypes using a wireless keyboard. The Vision for Action Test was performed in a dimly lit, sound-insulated testing room using an Eyelink 1000+ eye tracker and a Dell computer with a 24” 144 Hz LED monitor. A chinrest was used to control viewing distance from the screen (57 cm). Progress of the experiment was tracked from a separate room using two monitors, one to monitor and control the Eyelink 1000 Hz monocular eye-tracking system and one to monitor participants’ behaviour and adherence to instructions.

**Display and stimuli.** For testing visual acuity (Freiburg Vision Test), Landolt C stimuli were used. The optotype C was used at eight different orientations and was presented in black against a white background. The optotype was displayed at the centre of the screen and the contrast value of the optotype was set at 100%. A visual display monitor presented the optotypes with a viewing distance of 4 m. Pixel size was approximately 0.2 mm. The size of the optotype details (stroke width and gap width) subtended 1/5th of the overall height of the optotype. Cue stimuli for the Vision for Action Test comprised a red circle and a red triangle on either side of the central fixation cross. Each shape was 1.3° width and 0.8° height, presented 17° (from central fixation to the centre of shape stimuli) on either side of central fixation. Target stimuli for this task were the digits 2 or 7, subtending 0.5° width and 0.7° height and presented 17° to either the left or right of central fixation.

**Procedure.** In the Short Physical Performance Battery (SPPB; [34]), participants were timed while performing tasks that included standing with feet together and side by side, a semi-tandem stand and a tandem stand. Gait speed and repeated chair stands were also recorded. Participants were asked to walk at their normal pace across a 4 m room and were required to sit on a chair and stand up five times without using their hands. Times for each task were recorded and scored (1–12) according to the SPPB protocol (see [34] for full details). Higher scores indicate better performance.

### 5.1. Freiburg Vision Test (Visual Acuity)

A full description of the Freiburg Vision Test is provided by Bach [18]. Initially, participants were presented with a relatively large (0.83°) Landolt C visual stimulus. Participants were required to indicate the position of the gap in the optotype using the numbers in corresponding positions (1, 2, 3, 4, 6, 7, 8, 9) on the number pad of the keyboard. Hence, optotype stimuli were presented in eight alternative orientations (Guessing rate = 12.5%, see [18]). Depending on whether the response was correct or incorrect, an easier or more difficult to identify optotype was presented, in order to establish a visual acuity threshold for each participant. A forced-choice procedure was used; thus, participants were required to respond even if they were unsure of the position of the gap. The optotype remained on the screen until a response was made.

### 5.2. Vision for Action Test (VAT)

VAT administration comprised two blocks of the same procedure. The procedure began with an eye-tracker calibration, followed by task instructions. Participants were informed that one of the peripheral cue shapes was 100% predictive of subsequent target location, and that they were to look at the peripheral target (a digit) as fast as they could and then to respond via keypress whether the target had been a ‘2’ or a ‘7’ (down arrow or up arrow, respectively). The task began with a practice set of 10 trials followed by the full procedure containing a variable amount of trials, which concluded when a VAT threshold of 75% response accuracy was determined.

**Figure 1:**
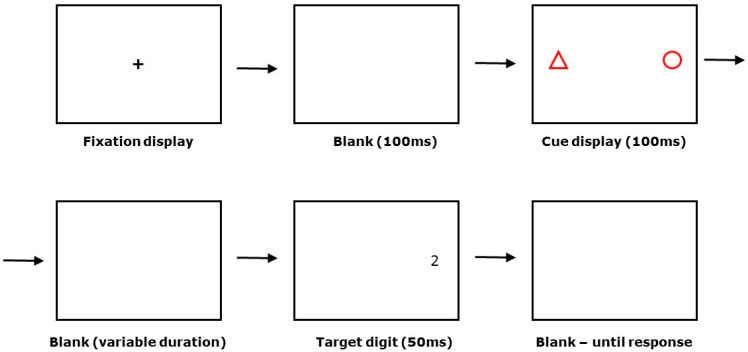
Structure of an individual trial in the Vision for Action Test (see text for further details).

The procedure followed on each trial is summarised and illustrated in Figure 1. Each trial began with a presentation of a central fixation cross for 1000 ms, followed by a blank screen for 100 ms, followed by re-presentation of the fixation cross for 1000 ms, followed by a blank screen for 100 ms. This was to ensure that participants were focusing on the centre of the screen at the beginning of each trial as they were instructed to do. This was followed by 100 ms of cue presentation, a variable blank stimulus onset asynchrony (SOA) screen, 50 ms of target presentation, and finally up to 1000 ms of blank screen awaiting target response. The initial SOA value was set to 1000 ms. This value was subsequently reduced in a staircase procedure [24] until the accuracy of the participant’s response was stable at 75% correct. The initial step size for reducing cue–target SOA was set at 10 ms. The staircase procedure exited when the number of ‘effective reversals’ in the value of the cue–target SOA equalled 4. In the procedure recommended by Findlay [24], the number of ‘effective reversals’ depends on a parameter M (see [24] for further details). For this study, the value of M was set at 4. If accuracy at the initial SOA value did not exceed 75%, the threshold value for that participant was recorded as 1000 ms. The target stimulus always appeared in the same location as one of the shapes; which shape was predictive was counterbalanced between participants. Post target offset, participants had up to 1000 ms to indicate whether the target was ‘2’ or ‘7’. A trial was scored as correct if a participant launched a saccade of 4° or more towards the landmark shape and target or they discriminated the identity of the target correctly by pressing the appropriate arrow key. Target responses were followed by a 1250-ms inter-trial interval before the next trial began.

## 6. Results (Stage 2)

Exploratory analysis of APQ scores and VAT threshold scores, our variables of central interest, revealed that VAT threshold scores deviated from normality (*p* = 0.046, Kolmogorov–Smirnoff test, with Lilliefors significance correction). This was remedied by applying a log transformation. After transformation, log VAT threshold scores did not deviate reliably from normality, *p* = 0.20. Our central prediction, that accident vulnerability will be related to vision for action, independently of vision for perception or age, was tested by evaluating the partial correlation between APQ scores and log VAT thresholds, controlling for visual acuity and age. This was statistically reliable, *r* = 0.71, df = 10, *p =* 0.005 (one-tailed). A bootstrap procedure (Bias-Corrected and accelerated—BCa), with 10,000 samples was used to establish 95% confidence intervals for this relationship: lower CI, *r* = 0.22; upper CI, *r =* 0.92. The relationship between APQ scores and log VAT thresholds remained reliable, when physical performance (SPPB) scores were added as a third control variable, *r* = 0.73, df = 9, *p* = 0.006 (one-tailed).

**Figure 2:**
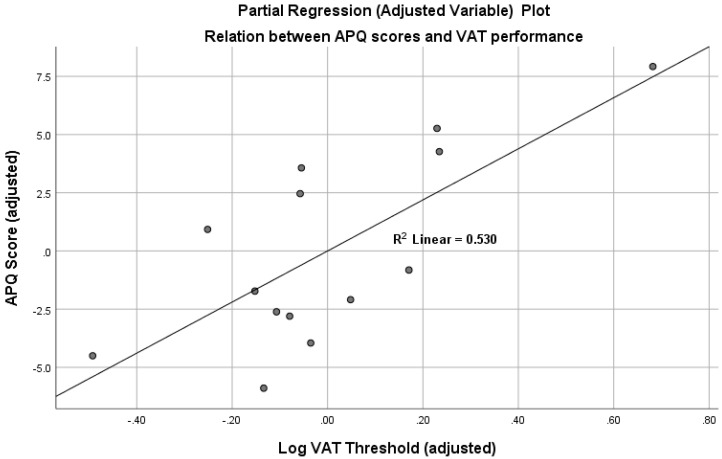
Partial regression (adjusted variable) plot, illustrating the relation between accident vulnerability (APQ scores) and individual differences in vision for action (log VAT thresholds), controlling for visual acuity (Freiburg Vision Test), age and gait performance (SPPB scores). (Note: plot exported from IBM SPSS Version 25, multiple regression output.).

The simple bivariate correlation of APQ scores with log VAT threshold was reliable, *r* = 0.51, df = 12, *p* = 0.033 (one-tailed). However, bivariate correlations of APQ scores with visual acuity (*r* = 0.09, df = 12, n.s.), APQ with age (*r* = 0.06, df = 12, n.s.) and APQ with SPPB scores (*r* = −0.10, df = 12, n.s.) all failed to approach significance. Multiple regression (IBM SPSS, Version 25) was then performed, with APQ scores as the dependent variable and log VAT thresholds, visual acuity, age and SPPB gait scores as predictors. As might be expected from the partial and bivariate correlations just described, the only reliable predictor of APQ scores was log VAT threshold, *t* = 3.19, *p* = 0.006 (one-tailed). This association is illustrated in Figure 2, which shows a partial regression (adjusted variable) plot of the relationship between accident vulnerability (APQ scores) and VAT performance, controlling for visual acuity, age and physical performance scores. Finally, participants were divided into high and low VAT threshold groups on the basis of median split. Analysis of covariance, controlling for age and visual acuity, showed that APQ scores were higher in the high threshold (adjusted mean = 22.90, 95% CI: 19.21 to 26.59) compared to low threshold (adjusted mean = 16.77, 95% CI: 13.08 to 20.46), *F*(1,10) = 5.50, *p* = 0.04.

## 7. Discussion

In agreement with our prediction, accident vulnerability was related to individual differences in vision for action, rather than individual differences in a conventional measure of vision reflecting vision for perception. Moreover, this relationship was independent of participant age and independent of a measure of physical performance (SPPB), which has been associated in previous work with accidental falls [35]. This finding reconciles the intuitive belief that visual failure appears to play a significant role in many accidents, with evidence that the association between accident risk and conventional visual assessments is weak or non-existent [7,8]. The current findings suggest a resolution to this conundrum—by taking seriously the distinction between vision for perception and vision for action, embodied in dual-stream models of vision motivated by findings from visual neuroscience [1,2]. Accordingly, it appears that accident vulnerability *is* related to visual functioning, but, in the current study, this association was only apparent in the link between APQ scores and our novel measure of vision for action. Individual differences in a conventional measure of vision for perception, the Freiburg Visual Acuity test, failed to predict accident vulnerability. Accident vulnerability was also unrelated to SPPB scores, a measure of the ability to carry physical actions that involve walking, standing and maintaining balance. Accordingly, one might summarise the study outcome in this way: accident vulnerability was unrelated to a conventional measure of vision (acuity), and unrelated to a measure of action (Short Physical Performance Battery), but was related to a measure that assesses the ability to link vision with action.

The issue of sample size and confidence deserves comment. Due to unavoidable time constraints associated with this project, data collection was terminated with a smaller-than-planned number of participants. The number of individuals who participated in the critical, second stage of the project was quite low (N = 14). Nevertheless, the predicted effect was highly reliable (*p* = 0.005), and the association between APQ scores and VAT thresholds, when controlling for age and acuity (*r* = 0.71) suggests a moderate-to-strong correlation. On the other hand, as one might expect, given the size of our sample, the confidence intervals associated with this correlation are wide, with a lower confidence limit (95%) of *r* = 0.26, corresponding to a weak relationship. In light of this, it seems appropriate to conclude that results from this initial test of our hypothesis provide very promising preliminary evidence that Vision for Action Test scores may have a useful predictive relationship with accident vulnerability. Further work with substantially larger sample sizes is planned, in order to establish with greater confidence the strength of the link between VAT performance and accident vulnerability.

The link identified in this study, between accident vulnerability and vision for action, may prove valuable in a variety of applied contexts. For example, the procedure embodied in VAT may prove useful when screening individuals for fitness to drive. As noted earlier, current visual assessments are essentially useless in this context [7,8]. VAT assessment may also be valuable in the context of intervention studies aiming to reduce accident vulnerability and improve quality of life in older adults. The health benefits of regular aerobic exercise are well-recognised [41,42]. However, it is conceivable that aerobic exercise that includes specific activities targeting eye-hand and eye-body coordination (e.g., ball games, dancing) could induce neuroplastic changes in dorsal stream regions and reduce accident vulnerability via improved vision for action. At the other end of the ability spectrum, it is possible that VAT assessment could be applied to the task of identifying individuals likely to display exceptional sporting talent, especially in fast-ball sports such as baseball, cricket or tennis.

While the results reported here indicate a positive relationship between vision for action and accident vulnerability, a variety of further questions can be asked, concerning the precise factors that drive this association. Although the ten items comprising the APQ all involve self-report of accidental events, the events in each question differed somewhat. Items 1–5 were concerned with falls, collisions and dropping objects, while items 6–10 were concerned specifically with accidents and near-misses on the road, as a pedestrian, driver, cyclist or motorcyclist. Although the internal consistency of the APQ was acceptable, given the small number of test items, (Cronbach’s alpha = 0.69, mean inter-item correlation, *r* = 0.17), further work will be needed to establish whether the measure is unidimensional, or composed of distinct factors, reflecting specific categories of accident, and, in the latter case, whether VAT performance is more strongly associated with particular kinds of accident.

In conclusion, we report promising initial evidence that accident vulnerability is related to vision for action, reflected in VAT threshold scores. Moreover, this relationship was independent of vision for perception (visual acuity), independent of participant age, and independent of the ability to perform physical actions involving balance, standing and walking. The procedure embodied in VAT may prove useful in a variety of applied contexts.

## Figures and Tables

**Table 1:** Mean APQ, FES-I and CFQ scores for younger and older adults.

Measure	Younger Adults (*N* = 15) Mean (SD)	Older Adults (*N* = 27) Mean (SD)	*t* (df)	*p*
**APQ**	20.79 (4.44)	17.76 (6.52)	1.60 (40)	n.s.
**FES-I**	1.746 (0.683)	1.599 (0.711)	0.65 (39)	n.s.
**CFQ**	40.80 (9.70)	33.52 (17.33)	1.50 (40)	n.s.

**Table 2:** Bivariate correlations (N) between APQ, FES-I and CFQ.

	FES-I	CFQ
**APQ**	0.492 ** (41)	0.529 ** (42)
**FES-I**		0.306 (41)

Note: ** *p* < 0.001.

## Data Availability

The data that support the findings of this study are openly available in Mendeley at **DOI:** 10.17632/7zkcbpc6cx.1. The name of the Mendeley dataset is: ‘Lambert, Sharma, Ryckman Accident vulnerability’.

## Appendix A

                **Accident Proneness Questionnaire**

                    **(Scoring Rubric)**

1. **Have you had a fall in the last year?**
           Yes (How many times) _________
           No
  (No = 1; Yes = 5)
2. **Have you ever required medical attention, as a result of having a fall?**
           Yes (How many times) _________
           No
  (No = 1; Yes = 5)
3. **Do you hurt yourself as a result of bumping into objects such as furniture, doors or lamp posts?**
           Very often
           Quite often
           Occasionally
           Very rarely
           Never
  (Never = 1; Very rarely = 2; Occasionally = 3; Quite Often = 4; Very often = 5)
4. **Have you ever required medical attention, as a result of bumping into something and hurting yourself?**
           Yes (How many times) _________
           No
  (No = 1; Yes = 5)
5. **Do you drop things you are holding such as your phone, cups or books?**
           Very often
           Quite often
           Occasionally
           Never
  (Never = 1; Very rarely = 2; Occasionally = 3; Quite Often = 4; Very often = 5)
6. *If your primary method of road transport is driving, please respond to this question; otherwise leave blank.*
  (a) **Within the past 5 years have you been involved in a road accident while driving?**
          Very often
          Quite often
          Occasionally
          Never
  (Never = 1; Very rarely = 2; Occasionally = 3; Quite Often = 4; Very often = 5)

*If your primary method of road transport is riding a motor bike, please respond to this question; otherwise leave blank.*

(b) **Within the past 5 years have you been involved in a road accident while riding a motorbike?**
           Very often
           Quite often
           Occasionally
           Never
  (Never = 1; Very rarely = 2; Occasionally = 3; Quite Often = 4; Very often = 5)

*If your primary method of road transport is riding a bicycle, please respond to this question; otherwise leave blank.*

(c) **Within the past 5 years have you been involved in a road accident while riding a bicycle?**
           Four or more times
           Three times
           Two times
           Once
           Never
  (Never = 1; Very rarely = 2; Occasionally = 3; Quite Often = 4; Very often = 5)

7. **Within the past 5 years have you been involved in a road accident as a pedestrian?**
           Four or more times
           Three times
           Two times
           Once
           Never
  (Never = 1; Very rarely = 2; Occasionally = 3; Quite Often = 4; Very often = 5)
8. *If you drive, ride a motorbike, or cycle on the roads please answer this question; otherwise leave blank.*
   **Do you sometimes fail to notice someone crossing at a pedestrian crossing, and keep driving/riding/cycling when you should have stopped?**
           Very often
           Quite often
           Occasionally
           Never
   (Never = 1; Very rarely = 2; Occasionally = 3; Quite Often = 4; Very often = 5)
9. *If you drive, ride a motorbike, or cycle on the roads please answer this question; otherwise leave blank.*
   **When turning at an intersection, do you sometimes fail to notice an approaching vehicle, and begin your turn when you should have waited?**
           Very often
           Quite often
           Occasionally
           Never
   (Never = 1; Very rarely = 2; Occasionally = 3; Quite Often = 4; Very often = 5)
10. **When crossing the road, do you sometimes fail to notice an approaching vehicle and begin to cross, when you should have waited?**
           Very often
           Quite often
           Occasionally
           Never
  (Never = 1; Very rarely = 2; Occasionally = 3; Quite Often = 4; Very often = 5)

**Note:** The ten items of the APQ were presented in the context of other accident-related questions that provided qualitative information (e.g., What were you doing mostly, prior to your fall/s? Walking; Exercising; Standing; Getting up from a chair; Getting out of bed; Other (please state); Not applicable—I rarely fall over).
